# The Story of the Insane from Year to Year

**Published:** 1902-08-30

**Authors:** 


### THE STORY OF THE INSANE FROM YEAR TO YEAR.

Fifteenth Annual Series.

### ROYAL ASYLUM, MONTROSE.

Here an increase of 42 patients has to be noted, and on the last day of the year there were 929 in residence. There were 194 first admissions, 68 readmissions, 121 discharged recovered, 15 discharged relieved, 25 not improved, and 59 deaths. The average number resident was 900, and the total number under treatment was 1,136. The recoveries stand at 46·1 per cent. of the admissions, and the deaths at 6·5 per cent. of the average number resident, the former being high and the latter low. Of the 59 deaths, 21 were caused by cerebral and spinal diseases, 5 by consumption, and 5 by pneumonia. Of the 262 admissions, 16 owed their insanity to various moral causes. Among the physical causes intemperance in drink accounts for 40, previous attacks for 47, and hereditary influence for 66. In 22 no cause was known. Dr. Reid describes at some length the history of one of the patients admitted during the year—a case of so-called impulsive mania. Seven years previously he had made an impulsive attack on a friend, and subsequently he had several minor seizures, and lastly he threatened to shoot two of his relatives and a police constable. The patient's own statement was that for an hour after these attacks he felt as if awakening from a sleep and retained only a vague idea of what had passed. Since being in the asylum he has been quiet and well-behaved in every way. But he has had one attack of momentary loss of consciousness, doubtless, as Dr. Reid says, minor epilepsy. Such cases are very interesting and instructive from a medico-legal standpoint, and they are frequently the subjects of criminal prosecutions. An expert examining such a patient in prison probably days, or it may be weeks, after the occurrence cannot be expected to find evidence of insanity, and he would not be allowed to state his opinion of the state of the prisoner's mind at the time of the homicidal attack. Seventeen of the staff entered for the nursing certificate of the Medico-Psychological Association, and all passed. Dr. Reid has had a trying time of it. His two assistant medical officers left, and 57 new attendants and nurses were engaged.

